# Supplementary material for: Rasagiline Inhibits Human Melanoma Cell Viability and Interacts Synergistically with Mitoxantrone and Antagonistically with Cisplatin—In Vitro Isobolographic Studies
Source: Cancers (Basel). 2025 Aug 3;17(15):2563. doi: 10.3390/cancers17152563 (PMC12345938; doi:10.3390/cancers17152563)
Supplement: Supplementary file 1 [file cancers-17-02563-s001.zip › cancers-3781835-supplementary.pdf]

Supplementary Materials:

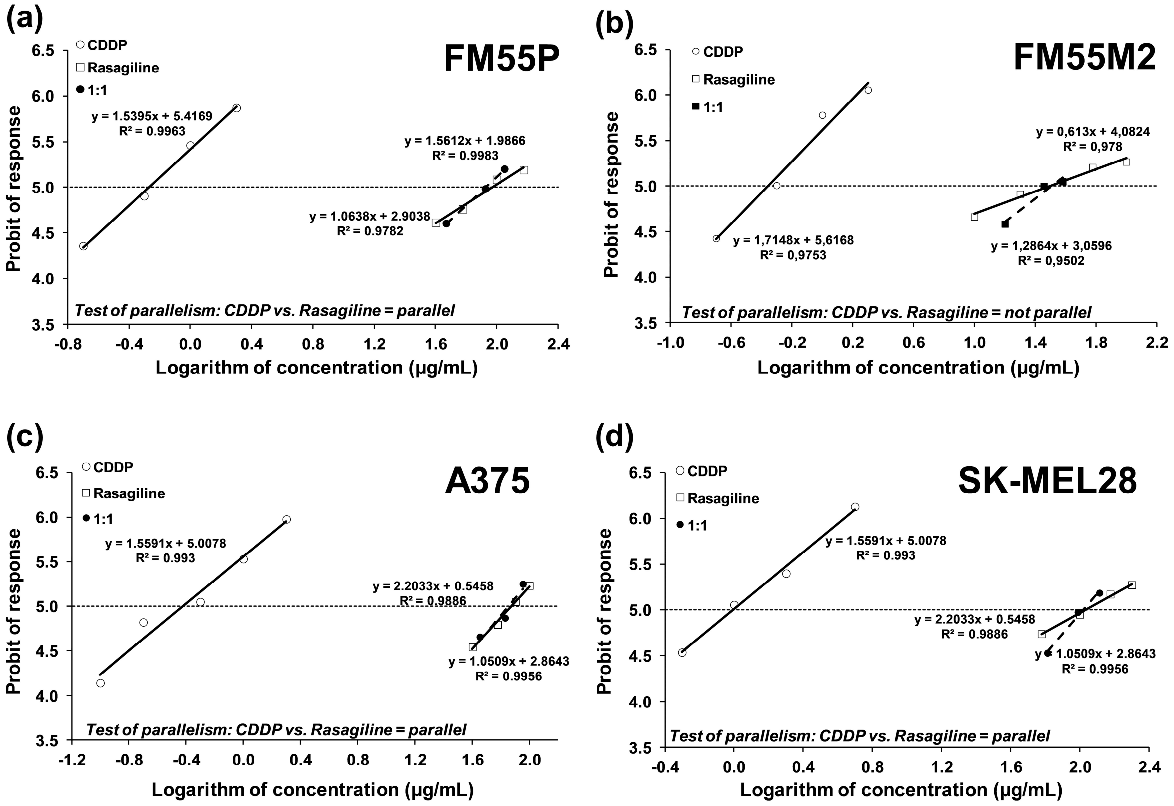

Figure S1: Log-probit concentration–effect lines for rasagiline and cisplatin (CDDP) administered alone and in combination in the fixed-ratio of 1:1, illustrating the anti-proliferative effects of the drugs in the malignant melanoma cell lines: FM55P (a), FM55M2 (b), A375 (c), and SK-MEL28 (d) measured in the MTT assay;

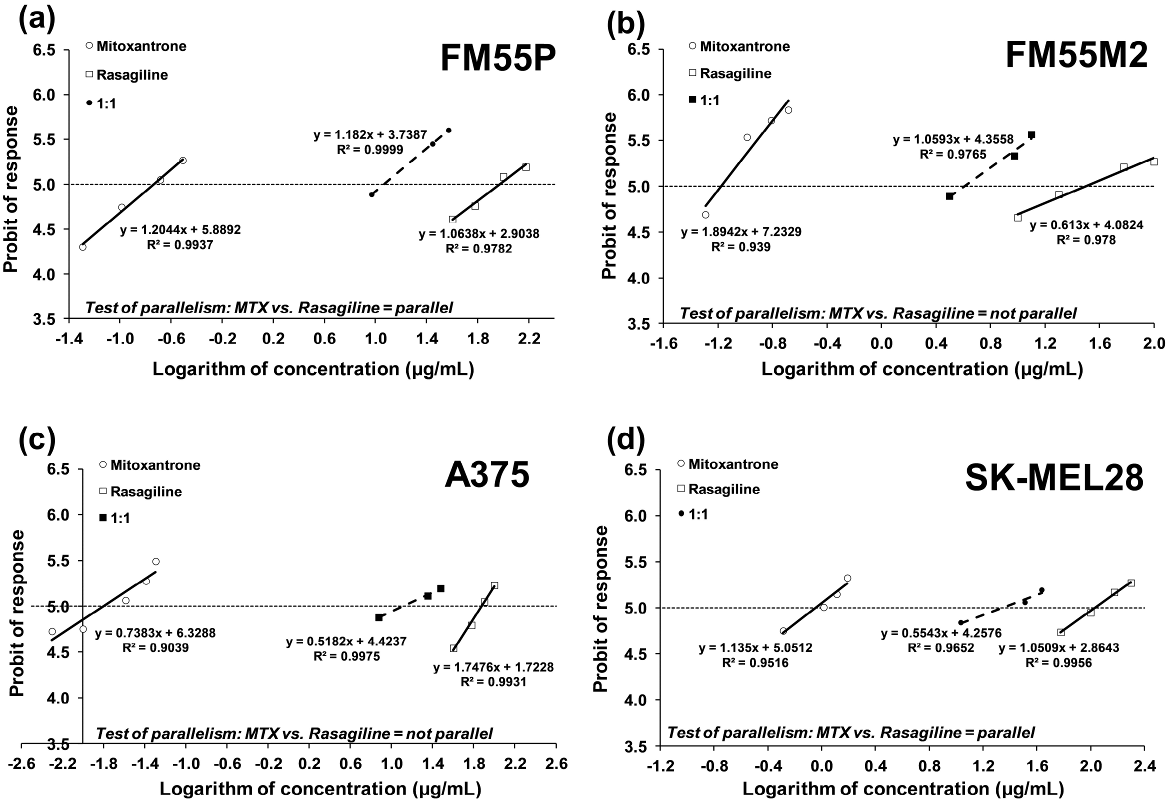

Figure S2: Log-probit concentration–effect lines for rasagiline and mitoxantrone (MTX) administered alone and in combination in the fixed-ratio of 1:1, illustrating the anti-proliferative effects of the drugs in the malignant melanoma cell lines: FM55P (a), FM55M2 (b), A375 (c), and SK-MEL28 (d) measured in the MTT assay.
